# Growth differentiation factor 11: a “rejuvenation factor” involved in regulation of age-related diseases?

**DOI:** 10.18632/aging.202881

**Published:** 2021-04-22

**Authors:** Yuting Ma, Yongping Liu, Fang Han, Hongyan Qiu, Junfeng Shi, Na Huang, Ningning Hou, Xiaodong Sun

**Affiliations:** 1 Department of Endocrinology, Affiliated Hospital of Weifang Medical University, Weifang, China; 2 Department of Clinical Research Center, Affiliated Hospital of Weifang Medical University, Weifang, China; 3 Department of Pathology, Affiliated Hospital of Weifang Medical University, Weifang, China

**Keywords:** growth differentiation factor 11, GDF11, aging, transforming growth factor, age-related diseases, regeneration

## Abstract

Growth differentiation factor 11 (GDF11), a member of the transforming growth factor β superfamily of cytokines, is a critical rejuvenation factor in aging cells. GDF11 improves neurodegenerative and neurovascular disease outcomes, increases skeletal muscle volume, and enhances muscle strength. Its wide-ranging biological effects may include the reversal of senescence in clinical applications, as well as the ability to reverse age-related pathological changes and regulate organ regeneration after injury. Nevertheless, recent data have led to controversy regarding the functional roles of GDF11, because the underlying mechanisms were not clearly established in previous studies. In this review, we examine the literature regarding GDF11 in age-related diseases and discuss potential mechanisms underlying the effects of GDF11 in regulation of age-related diseases.

## INTRODUCTION

Aging is currently a source of considerable concern worldwide. The prevalence of age-related diseases increases rapidly with advancing age; these diseases include cardiovascular disease, cognitive impairment, cancer, Alzheimer's disease, arthritis, obesity, and diabetes [1–3]. Aging has been linked to the progressive accumulation of damage and loss of function, both of which contribute to the onset of chronic disease and eventual death. Thus, there is ongoing research concerning the extension of healthy life and potentially reversing the aging process. Currently, senescent cells have become an increasingly important therapeutic target for age-related diseases [4]. Importantly, senescent cardiomyocytes contribute to cardiac fibrosis, while senescent neurons and glial cells led to neurodegenerative diseases [5]. Senescent cells exhibit various age-related characteristics, including irreversible cell cycle arrest, DNA damage, inflammation and oncogenes [6], resistance to apoptosis, and the acquisition of a senescence-associated secretory phenotype [7]. This phenotype involves the secretion of multiple signaling molecules, including transforming growth factor-β (TGF-β), which induce and maintain age-related pathological conditions [8].

TGF-β is a family of pleiotropic cytokines with more than 30 members; it includes growth differentiation factors, bone morphogenetic proteins, and activins [9]. These cytokines regulate multiple cellular biological procedures such as embryogenesis, homeostasis, and various pathological states [10, 11], implying a relationship between TGF-β signaling and the onset of age-related diseases. TGF-β signaling impairment and elevated TGF-β ligand concentrations in certain cell types may contribute to cell degeneration, inflammation, reduced regeneration ability, and metabolic abnormalities associated with age-related diseases [8].

Growth differentiation factor 11 (GDF11), a member of the TGF-β superfamily, has recently received attention because of its numerous functions in modulating the development and differentiation of various tissues and organs. It was initially identified by McPherron et al. as a new differentiation factor for odontoblasts [12]. Studies regarding the role of GDF11 in the development of various diseases have been conducted in recent decades. GDF11 is reportedly beneficial with respect to controlling age-related cardiac hypertrophy, improving muscle tone, preventing degeneration in the central nervous system, enhancing cognitive function, and promoting tissue regeneration [13, 14]. Important parabiosis experiments involving two animals of different ages, performed in 2013 and 2014, revealed that GDF11 levels were disrupted in an age-related manner in vascular, neurogenic, and skeletal muscle tissues [15, 16]. Those findings suggested that GDF11 may be regarded as an honorable “rejuvenation” factor that could restore regenerative function, thus resisting aging and extending longevity. A study in fish conducted by Zhou et al. revealed that GDF11 has rejuvenation capacity to extend the lifespan [17]. In 2020, a plasma proteomic dataset from Lehallier et al. demonstrated that the GDF11 protein can significantly extend the lifespan [18]. The above studies demonstrated critical roles for GDF11 in the inhibition of aging. However, recent studies have yielded conflicting data regarding the ability of GDF11 to alleviate dysfunction in age-related diseases [19, 20]. Thus, the regeneration ability of GDF11 with respect to age-related dysfunction requires further investigation. This review provides an overview of GDF11 and its functions in age-related diseases. It also discusses potential underlying mechanisms for the effects of GDF11 in age-related diseases.

## Structure and promotor of GDF11

**Figure 1:**
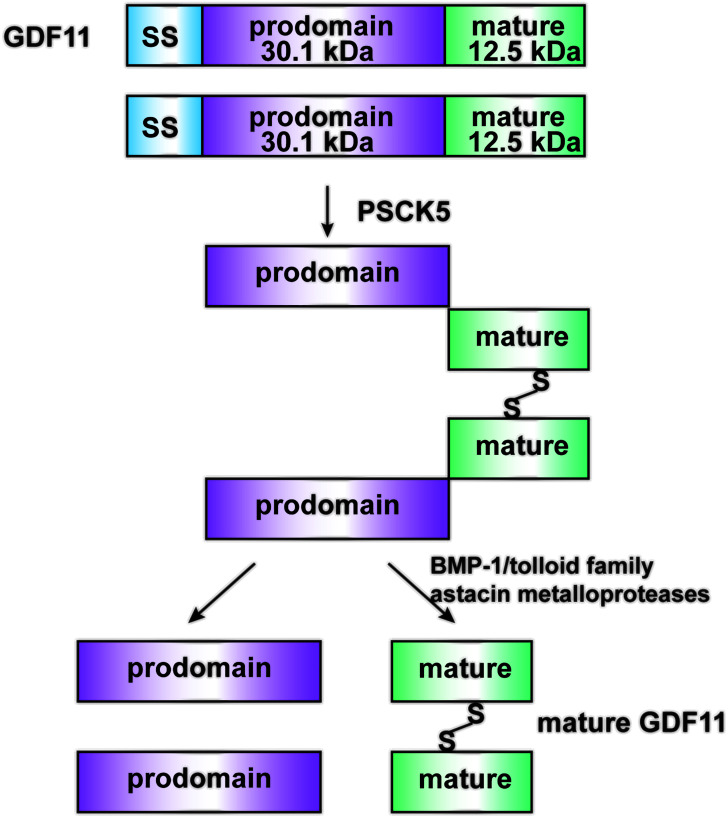
**Structure and Maturation process of GDF11.** GDF11 is cleaved by PCSK5 to form an inactive latent complex, which contains an N-terminal inhibitory pro-domain and two disulfide-linked carboxyl-terminal active domains. Then, members of the BMP1/Tolloid family of metalloproteinases cleave the latent complex at a single specific site to form the mature GDF11 and pro-peptide.

In humans, the *GDF11* gene is located on chromosome 12. The GDF11 protein comprises 407 amino acids; it contains a single peptide, an RXXR protein hydrolysis processing position, and a C-terminal domain with a highly conserved cysteine residue pattern [21]. Precursors of TGF-β-like proteins require cleavage at site 1 to release the mature portion of the growth factor [22]. The pro-protein convertase subtilisin/kexin 5 cleaves the GDF11 protein into an inactive latent complex, which contains an N-terminal inhibitory precursor domain and two disulfide-bonded active end domains [23, 24]. Bone morphogenetic protein-1/tolloid family astacin metalloproteases cleave the propeptide and activate non-covalently bound potential complexes, which are formed by the propeptide and mature protein dimers (disulfide-linked) in circulation [25, 26] (Figure 1).

**Figure 2:**
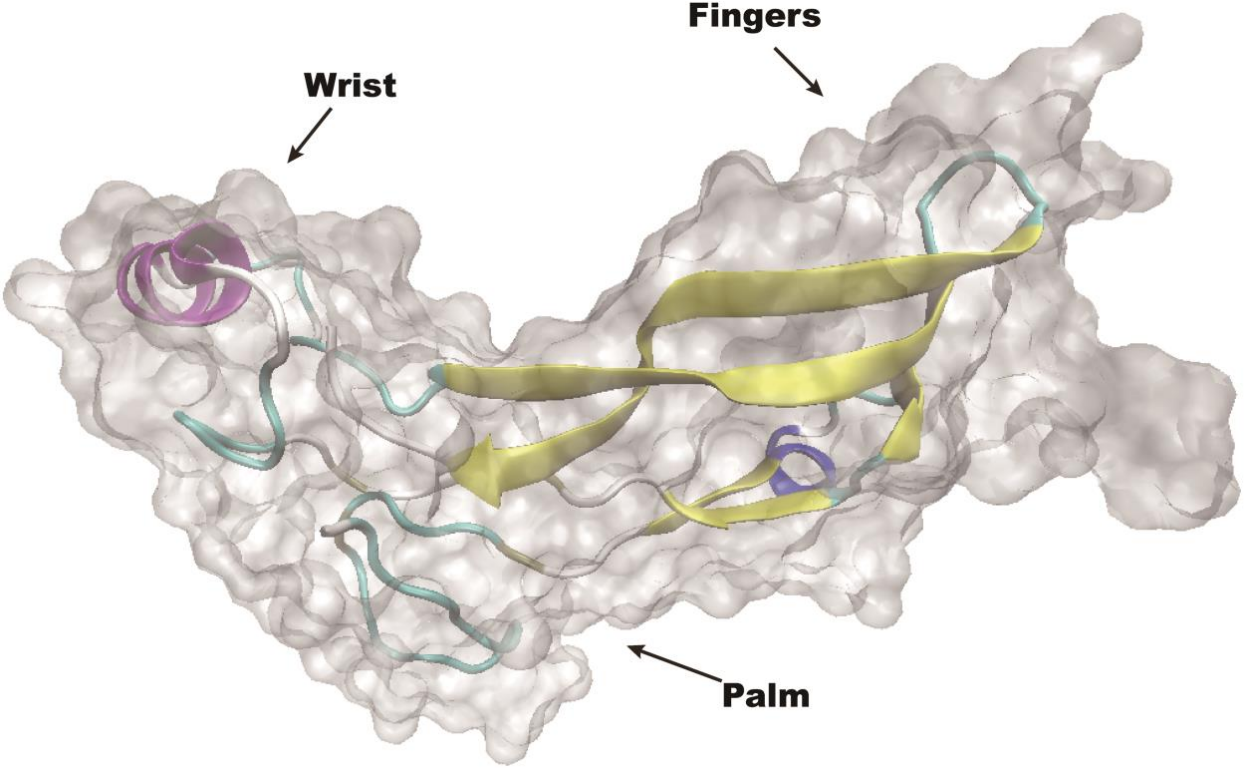
Overall structure of homodimeric human GDF11.

Crystallography analysis of GDF11 has revealed a standardized homodimeric form; monomeric GDF11 exhibits constitutive activity. The human GDF11 protein exhibits a conserved tertiary structure, similar to a “hand” with a four-stranded β-sheet that constitutes the “fingers,” as well as a cystine-knot structure that occupies the “palm” and an α-helix that forms the “wrist.” (Figure 2). The interlaced accumulation of adjacent dimers results in contact between the β-folded fingers of nearby molecules, as well as contact between the primary helix wrist of the homodimer chaperone and the β-sheet finger of the adjacent molecule [27].

The promoter regions of the *GDF* gene contain multiple E-box and ROR/REV-ERB response elements, which bind to many transcriptional activators to form a heterodimer that controls various downstream genes [28]. Two transcriptional products of the *GDF11* gene have been identified, according to Ensembl [29]. Despite GC enrichment (77%) in the promoter sequence of human GDF11, there are three well-concealed CCAAT boxes without a presumed stimulatory protein 1 site. These three CCAAT boxes are individually located at +87 bp and +171 bp (both downstream of the presumed transcription initiation point), and at -66 bp (upstream of the putative transcription start site). The CCAAT box at -66 bp is presumed to be sufficient and necessary for trichostatin A (TSA) to activate the GDF11 promoter [30]. TSA, an inhibitor of histone deacetylase 3 (HDAC3), is known to upregulate the expression of the gene encoding *GDF11* [30]. According to a comprehensive survey of human HDAC3, treatment of cells with TSA leads to the inactivation of HDAC3 and reduction of *GDF11* expression, revealing that HDAC3 is both necessary and sufficient for *GDF11* promoter activity [30]. A recent study suggested that the transcription factor zinc finger protein 740 (ZNF740) upregulates the hypoxia-induced expression of *GDF11* [31]. To verify the binding of transcription factors to the *GDF11* promoter, Yu et al. obtained information regarding *GDF11* promoter region transcription factors, including the nuclear factor of activated T cells 2, ZNF740, and specificity protein 1; these factors each target a separate motif [31]. ZNF740 is the only factor with an upstream site that is present in the *GDF11* initiation subsequence (-753/-744; CCCCCAC); it may participate in a growth factor pathway involved in the ZNF740/GDF11/Smad signaling axis [31].

## GDF11 signaling pathway

**Figure 3:**
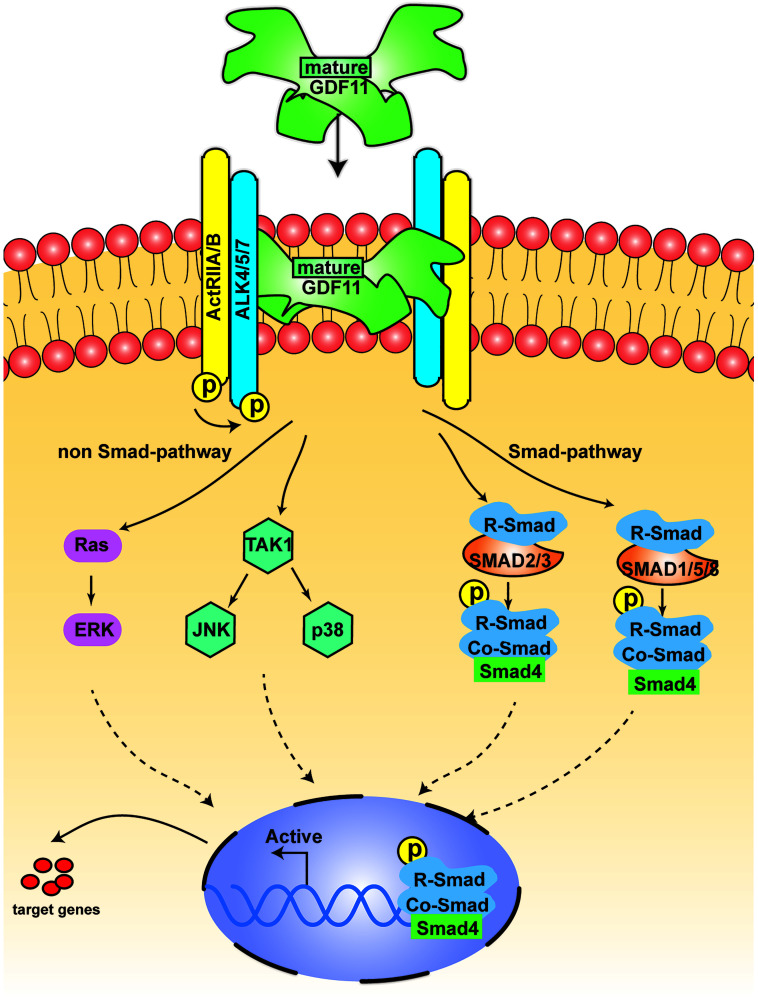
**The signal transduction of GDF11.** The figure displays the canonical signal transduction mediated by R-SMAD (SMAD 2/3, SMAD 1,5,8), assisted by the Co-SMAD (SMAD4). The non-canonical pathway is driven mainly by TGF-β activated kinase 1 (TAK1) and Ras.

Like other members of the TGF-β superfamily, GDF11 regulates cell signaling by binding to activin receptor types I (activin receptor-like kinase 4/5/7 [ALK4, ALK5 and ALK7]) and II (ActRIIA and ActRIIB) (Figure 3). Both types I and II receptors comprise a small extracellular ligand-binding domain and an intracellular kinase domain. Generally, type II receptors phosphorylate and activate type I receptors. The activated type I receptors then phosphorylate and activate the receptor-regulated SMAD dimer. This dimer recruits the co-SMAD, SMAD4, to form a trimeric complex, which eventually translocates to the nucleus and regulates gene expression [32]. Specifically, GDF11 binds to the ectodomains of the high-affinity type II receptor ActRIIB and the low-affinity type I receptor Alk5 to form a class of activin-type ternary complex crystals [33]. The ternary complex structure of GDF11/ActRIIB-ectodomain/Alk5-ectodomain then phosphorylates intracellular SMAD proteins. These SMAD proteins transduce the signal to the nucleus and act as transcription factors; thus, signal transduction outcomes are dependent on the ligand-receptor combination [33, 34]. There are two common SMAD signaling patterns, including the activation of SMAD 2/3 and SMAD 1/5/8 [35]. In addition to the typical SMAD signals, other non-SMAD pathways have been reported [19, 36]. GDF11 activates the adenosine monophosphate-activated protein kinase/endothelial nitric oxide synthase pathway, but suppresses the c-Jun amino-terminal kinase and NF-κB pathways. GDF11 can also activate p38 and extracellular signal-regulated kinase [19]. MitoTEMPO, a mitochondrion-targeted ROS inhibitor, inhibits the GDF11-induced activation of c-Jun amino-terminal kinase and adenosine monophosphate-activated protein kinase; thus, the GDF11-induced activation of c-Jun amino-terminal kinase and adenosine monophosphate-activated protein kinase can be modified by ROS status [37]. Recently, ERK1/2 signaling was found to be activated by GDF11, which downregulated bone morphogenetic protein–SMAD signaling and hepcidin activity [38].

## GDF11 is expressed in multiple tissues

**Figure 4:**
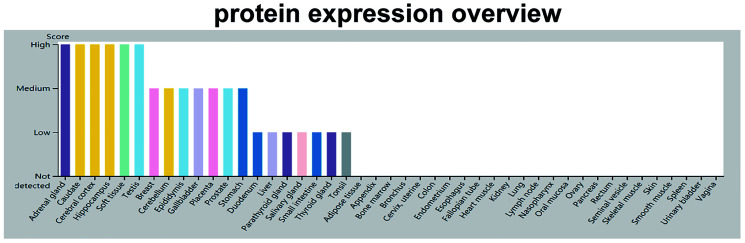
**GDF11 protein expression data.** The color-coding is based on tissues with common functional features. The mouse-over function shows protein score for analyzed cell types found in a selected tissue (**http://www.proteinatlas.org/ENSG00000135414-GDF11/tissue**).

After the initial discovery of GDF11 in odontoblasts, its distribution and expression were reported in other tissues [9]. Analysis of adult rat tissues revealed the expression of GDF11 in the skeleton, muscle, mandibular arch, hyoid arch, nasal epithelium, eye, spinal cord, olfactory system, kidney, testis, dental pulp, heart, brain, lung, spleen, and liver [9] (Figure 4). Notably, GDF11 was expressed in embryonic and adult brain regions: in various nuclei in the anterior hindbrain and ventral midbrain, as well as the thalamus, preoptic area, hippocampus, striatum, and outer layer of the inferior colliculus. In particular, GDF11 was strongly expressed in the thalamus and Purkinje cell layer, weakly expressed in the hippocampus, and inconsistently expressed in the midbrain and hindbrain [9]. Subsequently, GDF11 was expressed in the developing pancreatic epithelium, stomach, duodenum, and metanephros [39, 40]. Notably, GDF11 also comprises a circulating factor in blood [41]. However, there is inconsistency in the literature regarding circulating concentrations of GDF11 with age: reduction [42, 43], elevation [19] or tendency for elevation [44], or no change [42].

Additional contributors to the inconsistent conclusions include high structural homology between GDF11 and parabiosis resulting in difficulty distinguishing circulating GDF11 and GDF8, as well as the experimental contexts (e.g., serum sample manipulation, models, and assays to detect GDF11). These issues have been discussed exhaustively elsewhere [43, 45]. GDF11 has 90% amino acid sequence identity to GDF8 in its mature carboxyl-terminal domain. GDF8, also known as myostatin, is a specific negative regulator during skeletal muscle growth [12]. Rat *GDF11* has 88% identity to *GDF8* in the mature region [9]. However, the prodomains are only 52% identical between GDF8 and GDF11; these prodomains aid in the folding of mature dimeric ligand [46]. Because there is 90% sequence identity between mature active forms of GDF11 and GDF8, the SOMAmer technology and western blot analysis are not suitable assays for the recognition of GDF11 [19]. Importantly, Egerman et al. proposed an immunoassay that was specific for GDF11 and did not detect myostatin [19]. This immunoassay revealed elevated GDF11 levels in aged rats and humans; importantly, endogenous GDF11 could not be detected in young or old mice when it was below the detection threshold [19]. Katsimpardi et al. proposed another assay (sandwich ELISA) that demonstrated specificity for GDF11 by using recombinant myostatin, which was not detected at any concentration [43]; they also performed western blotting with an anti-GDF11 antibody that was fully validated for sensitivity and specificity to the GDF11 antigen [43]. Overall, the antibodies in these assays have contributed to differences in the results. We conclude that the reagent specificity and sensitivity are essential factors in determining the levels of GDF11. New and reliable studies can help move the field forward.

## GDF11 and age-related diseases

### GDF11 expression in cardiovascular disease

With the increasing prevalence of age-related heart failure worldwide, there is a critical need for adequate prevention and treatment methods [47]. Cardiac hypertrophy is a pathological feature of age-related heart failure. A recent study revealed that GDF11 could reverse age-related cardiac hypertrophy, implying an anti-hypertrophic role for GDF11 in age-related cardiac hypertrophy [41]. In that study, Loffredo *et al*. utilized parabiosis experiments involving young and old female C57BL/6 mice, in which the mice established a shared blood circulation with a “youthful” expression profile. The results showed that young circulation containing GDF11 reversed the aged hypertrophic cellular phenotype. These data and other *in vitro* evidence suggested that a “youthful” level of GDF11 in aged mice could reverse age-related cardiac hypertrophy [41, 48]. Adeno-associated virus carrying GDF11 protected against endothelial injury and attenuated atherosclerotic lesion formation both *in vivo* and *in vitro*, implying the potential for beneficial effects of GDF11 in the context of age-related cardiovascular diseases [49]. In the Heart and Soul study, GDF11 levels decreased in older participants, while the levels of GDF11 were associated with left ventricular hypertrophy and cardiovascular outcomes (e.g., death) [50]. To determine whether GDF11 directly prevents heart hypertrophy, the α1-adrenergic receptor agonist phenylephrine was used to promote hypertrophy of neonatal cardiomyocytes *in vitro* [51]. The results suggested that GDF11 prevents hypertrophy through the modulation of Ca2^+^ signaling and the Smad2/3 pathway in cultured neonatal rat ventricular myocytes treated with norepinephrine or phenylephrine [51, 52]. Those findings indicated that circulating GDF11 directly protects cardiac myocytes, consistent with its role in the prevention of excess hypertrophy [51, 52]. And also GDF11 protects against hypoxia-mediated apoptosis in cardiomyocytes by enhancing autophagy [53]. GDF11 inhibits cardiomyocyte pyroptosis in acute myocardial infarction mice [54]. Overall, circulating GDF11 has been shown to improve the progression of age-related cardiac hypertrophy, suggesting a novel treatment for this condition. However, previous studies regarding GDF11 in normal and diseased hearts have yielded disparate findings [55]. Recently, the presence of GDF11 at youthful levels, administered through injections of recombinant GDF11 (rGDF11), was not found to affect heart weight in 2-year-old C57BL/6 mice [20]. Furthermore, Smith et al. found that GDF11 did not rescue age-related pathological hypertrophy in 24-month-old C57BL/6 male mice, while Loffredo et al. reported the beneficial effect of rGDF11 in 23-month-old female mice. Notably, these studies used different types and sexes of mice. Loffredo et al. also implied that a reversal of cardiac myocyte hypertrophy is sex-independent. *In vivo* analyses of cardiac and skeletal muscle following the administration of excess GDF11 (i.e., bioactive GDF11 at supraphysiological levels) revealed unsatisfactory findings, including compensatory regeneration, skeletal muscle loss, cardiac dysfunction, and death [56, 57]; these results suggested that the administration of GDF11 at supraphysiological levels may cause damage. Therefore, GDF11 may be beneficial and serve as a promising therapeutic rejuvenation factor in age-related cardiovascular disease when its levels are appropriate.

### GDF11 expression in neurological disease

Declining neurogenesis and cognitive function with age are associated with lower numbers of neural stem cells, diminished remyelination, and reduced blood flow [58, 59]. Aging stem cells exhibited regenerative potential upon exposure to a young environment, while young stem cells lost their regenerative potential upon exposure to an aged environment [60]. Moreover, exposure to a youthful systemic environment promotes remyelination in aged animals [58]. Contextual fear conditioning, spatial learning, and memory skills were reportedly impaired when young mice were exposed to an aged systemic environment or plasma from an aged animal [59, 61]. These findings confirmed that blood-borne systemic factors could inhibit or enhance the growth of neural tissue in an aged environment. Importantly, Katsimpardi et al. generated heterochronic parabiotic pairs and found that youthful circulating factors can restore the self-renewal and differentiation potential of aged subventricular zone neural stem cell and stimulate endothelial cell proliferation by 88% compared to old serum. Then, treating endothelial cells with rGDF11 increased their proliferation by 22.9% compared to controls. These findings imply that GDF11 improves vascularity and blood flow in the neurogenic niche, thereby enhancing neurogenesis [15]. Additionally, the different distributions of circulating GDF11 between young and old brain tissues suggests that GDF11 may have crucial functions for neurons; it may provide novel approaches for the treatment of age-related neurological diseases (e.g., neurodegenerative and neurovascular diseases) [62]. For example, aging may cause a decline in hippocampal neurogenesis [63]; the injection of GDF11 enhances neurogenesis and increases neuronal activity in the hippocampus of 22–23-month-old mice [64].

Stroke has been reported to induce angiogenesis in the area surrounding the infarction. Furthermore, angiogenesis has been reported to deliver growth factors/chemokines to facilitate the migration of nerve cells and the survival of new neurons, which implies a strong relationship between angiogenesis and neurogenesis [65]. Therefore, angiogenesis is a crucial target for stroke treatment. The proliferation and angiogenesis of neuronal precursor cells were improved through the TGF-β/Smad2/3 signaling pathway following the injection of rGDF11 into stroke models [66]; thus, mice treated with rGDF11 exhibited remarkable enhancement of neuronal regeneration and functional restoration [66]. A recent study highlighted that GDF11 can reduce gliosis, improve angiogenesis, and attenuate the proliferation of glial cells after transient ischemic stroke in 20–22-month-old male mice with middle cerebral artery occlusion [67]. GDF11 has been proposed to improve nerve function recovery after ischemia/reperfusion damage to the brain; this action may be partly mediated by the onset of angiogenesis in the peri-infarct cerebral cortex, in association with ALK5 [68]. Thus, GDF11 and ALK5 may constitute novel therapeutic targets for stroke rehabilitation.

Alzheimer's disease is a complex heterogeneous disease that is caused by genetic, neurotransmitter, immunological, and environmental factors [69]. Nearly 90% of affected patients have cerebral amyloid vascular disease [70], which is characterized by the accumulation of β-amyloid peptide within the brain, as well as hyperphosphorylated tau protein [71]. In patients with Alzheimer's disease, large amounts of β-amyloid peptide are generated from amyloid precursor protein and accumulate in the brain, leading to acute neuronal toxicity [72] and synaptic dysfunction [73]. Classical neuropathology may exacerbate cognitive decline. Sub-chronic treatment of 12-month-old AβPP/PS1 mice (an animal model of Alzheimer's disease) with GDF11 has been shown to restore cognitive function and improve cerebrovascular function [74]. To further assess whether GDF11 can protect against the age-related progression of cognitive dysfunction, the levels of GDF11 were measured in human plasma from healthy adult men, healthy aged men, and aged men with distinct extents of age-related cognitive impairment. Notably, no relationships were found between age-related changes in circulating GDF11 levels and cognitive impairment, which suggested that circulating GDF11 may not be protective of cognitive function during aging [75].

Excess ROS in the brain has also been reported to contribute to human aging. The control of neurovascular units is reportedly dependent on the regulation of ROS levels. Excessive ROS levels can cause neuronal functional decline [76]. Many longevity-related signaling pathways are presumed to have essential roles in brain function, such as Forkhead box class O transcription factors and Sirtuin-1 [77, 78]. The manipulation of signaling molecules that affect Forkhead box class O and Sirtuin-1 activity has been shown to improve the ability of neurons to respond to ROS stress and can extend their overall lifespan [79]. There is evidence that GDF11 has a direct biological effect on capillary endothelial cells in the brain, based on the activation of TGF-β signaling following injection of rGDF11; thus, GDF11 may serve as a promoter of neurogenesis and angiogenesis [15, 80].

### GDF11 expression in skeletal muscle disease

Reduced skeletal muscle mass, strength, and physiological endurance are features characteristic of aging [81]. Aged muscle contains fewer numbers of satellite cells, exhibits impaired satellite cell function, and has low regenerative potential [82]. Therefore, the elevation of satellite cell numbers and improvement of satellite cell function are potential approaches for alleviating the effects of aging in muscle.

As a “young” circulating factor, GDF11 has been shown to restore skeletal muscle function, improve muscle structure, and enhance muscle strength and endurance exercise capacity in aged animals [16]. However, GDF11 has also been shown to reduce satellite cell expansion and significantly inhibit muscle regeneration by blocking myoblast differentiation via SMAD2/3 phosphorylation, p38 and ERK activation, and downstream signaling regulation [19]. Furthermore, GDF11 causes reductions of mass and function in both heart and skeletal muscle of mice treated with GDF11-secreting cells [57]. The relationship between GDF11 and skeletal muscle regeneration in aged rats was explored using a complex rat skeletal muscle injury model [83]. The results indicated that GDF11 treatment caused considerable enhancement of tissue fibrosis, accompanied by the reduction of functional recovery [83]. These results implied that the effects of GDF11 may be less beneficial than expected.

The roles of GDF11 in cardiovascular, neurological, and skeletal muscle diseases are confusing, presumably because of tissue-specific or species-specific differences in its expression [45]. GDF11 levels in different mouse strains are presumably affected by their genetic backgrounds [84]. Furthermore, a positive quadratic correlation was found between GDF11 and the mid-life-span of a mice strain, such that higher GDF11 levels were indicative of longer lifespans and might influence experimental results [84]. There are important limitations in the treatment of age-related diseases with rGDF11. First, lot-to-lot variability is evident in commercially available rGDF11 products. Second, there are differences in methods of administration, as well as bioavailability and dosing. Various circulating and tissue-specific factors may alter the effects of GDF11 or reduce its bioavailability [85]. In particular, GDF11 has a close association with myostatin, so high-quality and accurate assays are needed to distinguish these proteins. Third, mice used in some published studies were younger than in others, which may have introduced bias in the results. Finally, no prior studies have examined the potential for compensatory regulation of endogenous GDF11. Circulating GDF11 has minimal physiological relevance because it presumably cannot outcompete myostatin for ActRIIB binding sites [86]. In summary, the roles of circulating GDF11 in aging muscle, heart, and brain phenotypes should be reconsidered (Table 1).

**Table 1:** Effects of GDF11 in cardiac, muscle skeletal and nervous system disease.

**Age- related disease**	**outcome**	**overall effect**	**model**	**Study**
cardiac hypertrophy	positive	reversed age-related hypertrophy	heterochronic parabiosis	Loffredo FS, et al. [41]
atherosclerosis	positive	improve endothelial dysfunction, decrease endothelial apoptosis, reduce inflammation, decrease atherosclerotic plaques area	apoE^−/−^ mice	Mei W, et al. [49]
stable ischaemic heart disease	positive	lower risk of cardiovascular events and death	prospective cohort study	Olson KA, et al. [50]
pathological cardiac hypertrophy (PCH)	no	no effect on cardiac structure or function	C57BL/6 mice	Smith SC, et al. [20]
Cardiac disease	negative	decreased cardiomyocyte size and decreased cardiac function	Male athymic nu/nu mice	Zimmers TA, et al. [57]
the central nervous system	positive	rejuvenating synaptic plasticity and improving cognitive function	parabiosis pairs	Villeda SA, et al. [59]
stroke	positive	promoted neurogenesis and angiogenesis and contributed to functional recovery	8–10 weeks old male C57BL/6 mice	Lu L, et al. [66]
stroke	positive	improves neurofunctional recovery	cerebral ischemia/reperfusion (I/R) rat	Ma J, et al. [68]
Alzheimer's Disease	positive	restore cognitive function and improve cerebrovascular function	AD model mice	Zhang W, et al.[74]
ageing cognitive disease	no	may not exert a protective effect	prospective cohort study	Yang R, et al. [75]
muscle disease	negative	significant increase in tissue fibrosis, accompanied by attenuated functional recovery	complex rat model of skeletal muscle injury	Zhou Y, et al. [83]
muscle disease	positive	improved muscle structural and functional features and increased strength	heterochronic parabiosis	Sinha M, et al. [16]

Summary of the specific effects of increased GDF11 activity on cardiac, muscle skeletal and neurofunctional regeneration. Although the initial report suggested that GDF11 may have a rejuvenating function, follow up studies demonstrated an opposite, or negative effect.

### GDF11 expression in other age-related diseases

Many studies have explored the relationships of GDF11 with obesity and diabetes. Early reports showed that patients with type 2 diabetes or obesity exhibited higher circulating levels of GDF11 [49, 87], while others showed that circulating GDF11 levels were unaffected by the presence of obesity or type 2 diabetes [42]. Notably, GDF11 has been used to treat metabolic diseases, including obesity, insulin resistance, fatty liver development, and hyperglycemia. These outcomes suggest that GDF11 may be effective in the treatment of metabolic diseases [88, 89]. However, a new study showed that GDF11 may contribute to pathological fibrogenesis in a mouse model of non-alcoholic steatohepatitis [90]. There is some controversy regarding the roles of GDF11 in cancer biology. A recently published study indicated that GDF11 mediated tumor suppressor effects in triple-negative breast cancer [91], liver cancer [92], and pancreatic cancer [93]; however, an opposite effect was observed in colorectal cancer [94]. Additionally, some reports have demonstrated the pro-tumorigenic properties of GDF11 in oral squamous cell carcinoma [95, 96]. In patients with liver fibrosis and mouse models of experimentally induced liver fibrosis, observations regarding the upregulation of GDF11 expression imply that therapeutic application of GDF11 may resist fibrosis onset [97].

A positive correlation has been observed between the serum concentration of GDF11 and the level of thyroid-stimulating hormone [98]. The relationships of GDF11 with skin components have been evaluated in various skin models. Notably, enhancement of physiological GDF11 levels (via rGDF11 administration) led to the production of collagen I and hyaluronic acid in those models, along with the reduction of melanin, indicating potential benefits of GDF11 with respect to skin biology [99]. Aplastic anemia is a disease often characterized by bone marrow failure and pancytopenia. GDF11 levels are negatively correlated with hemoglobin levels in patients with aplastic anemia, suggesting a reduced response to GDF11 in these patients. Significantly higher GDF11 levels have been observed in patients with aplastic anemia [100]. Accordingly, the role of GDF11 in aplastic anemia requires further investigation.

## CONCLUSIONS

In this review, we described the gene structure and signaling pathways of GDF11, as well as the roles of GDF11 in organ development, aging, cardiovascular disease, neurological disease, and other diseases. Notably, GDF11 exhibits extensive expression in multiple tissues. Because of differences in GDF11 expression and function in cardiac, neural, muscular, and other tissues, further research is needed to elucidate the roles of GDF11 in age-related diseases. Current theories suggest that various rejuvenation factors in young blood have beneficial effects on cognitive and cardiovascular functions; the presence of GDF11 in many pro-longevity signaling pathways indicates that it may possess an ancient role in the regeneration of organ function. In this review, we have emphasized that the “youthful” expression of GDF11 (demonstrated via parabiosis experiments) may have a beneficial function in age-related diseases. Therefore, GDF11 may serve as a promising therapeutic rejuvenation factor in age-related diseases when its levels are appropriate.
